# Open and Minimal Approaches to Pancreatic Adenocarcinoma

**DOI:** 10.1155/2020/4162657

**Published:** 2020-05-27

**Authors:** Ricky H. Bhogal, Stephanos Pericleous, Aamir Z. Khan

**Affiliations:** Department of Surgery, The Royal Marsden Hospital, London. SW3 6JJ., UK

## Abstract

Surgical options and approaches to pancreatic cancer are changing in the current era. Neoadjuvant treatment strategies for pancreatic cancer combined with the increased use of minimal access surgical techniques mean that the modern pancreatic surgeon requires mastering a number of surgical approaches with to optimally manage patients. Whilst traditional open surgery remains the most frequent approach for surgery, the specific steps during surgery may need to be modified in light of the aforementioned neoadjuvant treatments. Robotic and laparoscopic approaches to pancreatic resection are feasible, but these surgical methods remain in their infancy. In this review article, we summarise the current surgical approaches to pancreatic cancer and how these are adapted to the minimal access setting with discussion of the patient outcome data.

## 1. Introduction

Pancreatic adenocarcinoma (PDAC) is one of the leading causes of death with 432,242 deaths per year and 458,918 new cases recorded per year according to GLOBOCAN 2018 [1]. Despite significant progress in the detection and management of PDAC, the 5-year survival is still reported at a disappointing 9% [2]. Surgical resection is the only treatment option that offers the prospect of long-term remission; however, only approximately 20% of those patients presenting PDAC are eligible for resection [3]. Surgery for PDAC has evolved since Allen Whipple reported the first surgical series in 1935 [4] with recent studies reporting an improved 5-year survival of 30% [5]. Despite this progress in improving patient outcome following surgery for PDAC, the modern pancreatic surgeon is faced with a number of new challenges that can involve modifications to the traditional surgical approach. These include the advent of neoadjuvant regimens to PDAC such as those in the current ESPAC-5 trial [6]. In addition, surgical advancements in minimal access and robotic surgery have added further techniques to the pancreatic surgeon's armamentarium. In this review, we summarise the current surgical approaches available for the pancreatic surgeon when planning surgery for patients with PDAC.

## 2. Current Era of Pancreatic Surgery for PDAC

Approximately 65% of PDAC is located in the pancreatic head with 15% in the body or tail and multifocal disease accounting for the remaining cases [5]. Indeed, an inferior survival is noted in patients with PDAC located in the body and tail relative to the pancreatic head [6], and as discussed below, this may be accounted for by the extent of lymphadenectomy performed in distal pancreatectomy. Therefore, after appropriate preoperative staging and investigations, patients with PDAC are generally eligible for one of three operations: Whipple/pylorus preserving pancreaticoduodenectomy (PPPD), total pancreatectomy with or without splenectomy, or distal pancreatectomy with or without splenectomy (DP). PPPD in contrast to the classical Whipple procedure retains the distal stomach and pylorus with the aim of decreasing the postoperative incidence of delayed gastric emptying, marginal ulcerations, and bile reflux gastritis, but the data to support this surgical approach is lacking [7]. In the modern era, all surgical approaches to the pancreas can be performed via the open, laparoscopic, or robotic routes. For instance, PDAC limited to the pancreatic head with no vascular involvement would be suitable of Whipple/PPPD whilst multifocal disease would be suitable for total pancreatectomy with splenectomy. There are now guidelines available on many aspects pertaining to pancreatic surgery for PDAC including the definition of standard lymphadenectomy, consensus statements of borderline resectable PDAC, and extended pancreatic resections, and the reader is referred here (https://www.ihpba.org/183_Guidelines-.html) for a further detailed information.

These international standardized nomenclatures for pancreatic surgery have allowed some degree of harmonisation in surgical approaches to PDAC and in time will also allow surgical data globally to be interpreted appropriately [8]. This is particularly important in an era of increased use of preoperative chemotherapy and/or chemoradiotherapy for PDAC in the presence of locally advanced disease (see below). The use of the neoadjuvant regimens means that the modern pancreatic surgeon must be prepared to adapt surgical approaches to achieve oncological pancreatic resections. To highlight this currently, patients with early-stage PDAC and no vascular involvement can be offered robotic pancreatic PPPD/Whipple (RPD) [9], whilst those surgeons further along the learning curve with laparoscopic approaches can offer laparoscopic PPPD/Whipple (LPD) with concomitant vascular resection [10] whilst patients receiving neoadjuvant chemoradiation will generally have open pancreatic resection [11].

As eluded to above, vascular involvement of either the superior mesenteric vein (SMV) and/or artery add an extra layer of complexity to surgical resection for PDAC. These patients are referred to as having “borderline resectable pancreatic cancer” (see below). Generally, these patients have limited vascular involvement with predominantly SMV involvement. A recent systematic review concluded that the portal vein and/or SMV resection combined with pancreatectomy is a safe and feasible, enabling an increased number of patients to undergo curative resection and improving patient survival [12]. However, in contrast, a recent meta-analysis suggests that patients undergoing vein resection as part of pancreatic resection have a higher R1 rate, lower survival, and the surgery was deemed not cost-effective [13], although a UK nationwide study has demonstrated that pancreatic resection combined with vein resection is superior to surgical bypass [14]. However, these data must be interpreted with caution; as surgical units comprising both hepatobiliary/transplant units, there is an increased likelihood of performing vascular resection due to the availability of cadaveric vein for portal vein/SMV reconstruction [15]. Whether cadaveric vein is used or another suitable conduit, the literature does support the use of vascular resection, especially venous resection, in PDAC to improve patient survival especially when neoadjuvant regimens have been utilized [16].

In summary, although international guidelines are available for the management of patients with PDAC, the optimal surgical approach in an era of increasing availability of surgical approaches and evolving neoadjuvant regimes means that constant reevaluation is required to ensure that patients receive optimal management. In the following section, we discuss the current surgical strategies available for the management of patient with PDAC.

## 3. Open Pancreatic Surgery with No Vascular Involvement

All patients with PDAC should have appropriate preoperative work-up with cross-sectional imaging and multidisciplinary team discussion prior to surgery. At present, most pancreatic surgeries are performed by surgeons specialising in hepatobiliary and pancreatic surgery at centralized tertiary referral centers, although data demonstrates that there is a marked global variation in the specialty of surgeons carrying out pancreatic surgery [17]. As discussed above, conventional Whipple or PPPD is considered for PDAC limited to the pancreatic head. In those patients with tumours confined to the pancreatic neck, a central pancreatectomy can be considered and if such tumours are extending toward the body/neck, then subtotal pancreatectomy can be performed in order to preserve pancreatic function [18].

**Figure 1:**
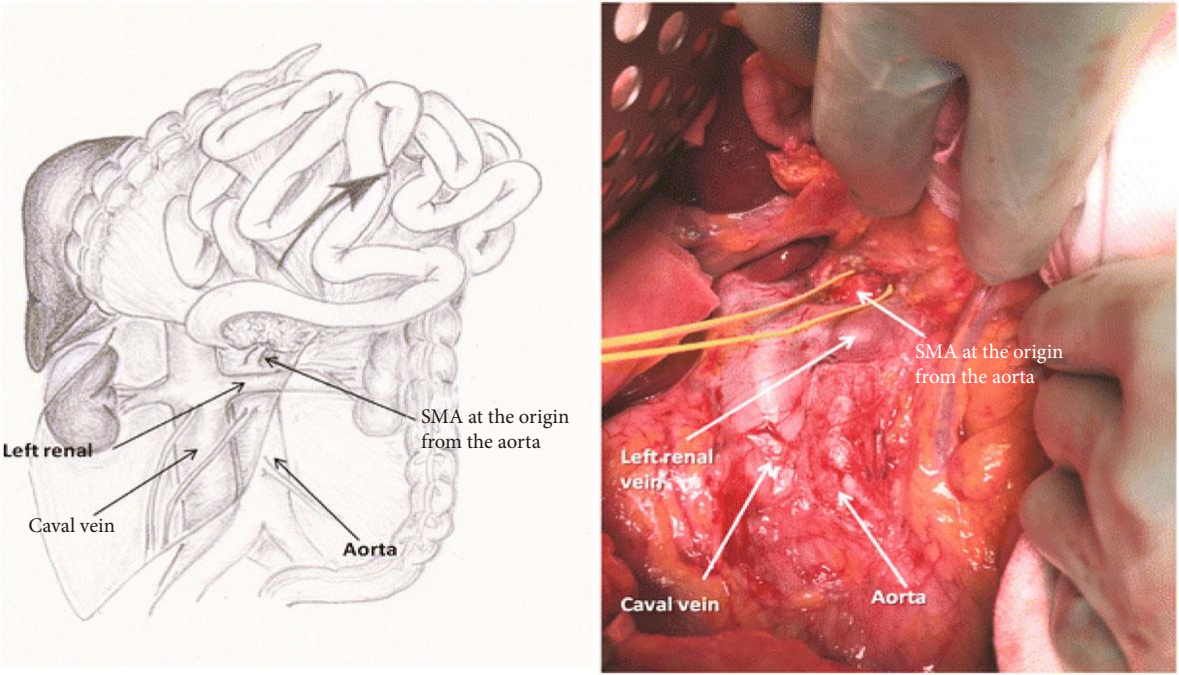
The Cattell-Braasch manoeuvre. The right colon, small bowel mesentery root, and duodenum (Kocherization) are mobilised from right to the left quadrant to allow exposure of the retroperitoneal structures and major vasculature. With completion of the Cattel-Braasch manoeuvre, the root of the SMA can be dissected free and SMA isolated from the tumour. (Taken from https://media.springernature.com/original/springer-static/image/art:10.1007/s11605-015-29581/MediaObjects/11605_2015_2958_Fig2_HTML.gif.)

A standard protocol for open pancreatic resection has not yet been internationally agreed. Therefore, in many instances, open pancreatic resection is performed based upon surgeon preference and patient factors. The generic oncological principles of resection, however, remain those of safe dissection, and appropriate and diligent lymphadenectomy to obtain an R0 resection. Surgeons may elect to perform Whipple/PPPD via a midline laparotomy or rooftop incision. The falciform ligament is divided and mobilised ensuring that it remains intact (see later). For patients in whom vascular resection is contemplated, the authors suggest a midline incision to enable the Cattell-Braasch manoeuvre to be performed (Figure 1). A thorough examination of the peritoneal cavity and liver is performed to exclude metastatic disease which would preclude resection. This may involve intraoperative ultrasound with some evidence to suggest that it may select out a small proportion of patients with liver metastasis [19]. After inspection of the peritoneal cavity, the surgeon may elect to perform a Cattel-Braasch manoeuvre and then adopt an “artery-first approach” (discussed below) or alternatively, the SMV may be approached by opening the gastrocolic ligament and dissecting it free from the inferior pancreatic border. In patients with disease limited to the pancreatic head, the Cattell-Braasch manoeuvre, which involves dissection along the right-sided white line of Toldt and then across the small bowel mesenteric root, allows the colon and small bowel to be retracted into the left upper quadrant and facilitates exposure of the SMV as it passes over the third part of the duodenum.

The complete extended Kocherization of the duodenum allows skeletonization of the inferior vena cava (IVC) and left renal vein. By using the superior border of the left renal vein as an anatomical landmark, the root of the SMA can be dissected free and neurectomy around the SMA can then be performed [20]. The Cattel-Braasch manoeuvre can be extended to the ligament of Treitz and with careful dissection the ligament can be fully mobilised and peritoneal cavity entered lateral to the ligament of Treitz. In the absence of neoadjuvant treatment, previous pancreatitis, or previous upper abdominal surgery, the Cattel-Braasch manoeuvre is achieved with minimal blood loss and economy of effort. As eluded to above, the Cattel-Braasch is recommended when the “artery-first approach” is being considered for resection [20]. The approach is discussed in detail below. In the conventional surgical approach, following Kocherization of the duodenum and skeletonization of the IVC and left renal vein, again the ligament of Treitz can be fully mobilised. The gastrocolic ligament can be opened widely with an energy device preserving the gastric arcade. The SMV can be identified by tracing the middle colic vein to the SMV at the lower pancreatic border. The retropancreatic tunnel can be developed at the inferior border of the pancreas. It is the author's view that if resistance is encountered here to suggest a portal vein/SMV involvement, then a Cattel-Braasch manoeuvre be performed at this point to aid vein reconstruction should it be required (see below).

**Figure 2:**
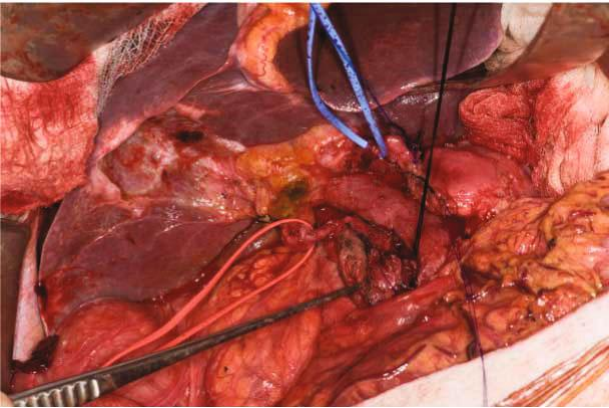
Preoperative CT triple phase may identify the anomalous anatomy shown here where the right hepatic artery originates from the SMA (red sloop) and left hepatic artery from the left gastric artery (blue sloop). The above patient had received neoadjuvant chemoradiation, and therefore, normal anatomical planes are not present and preoperative imaging can assist in safe dissection.

Following this, attention turns to dissection of the hepatoduodenal ligament. If the gallbladder is present, a cholecystectomy is performed with ligation and division of the cystic artery. The dissection is commenced at the base of segment 4 of the liver, and the peritoneum opened here. The left, right, and hepatic arteries are skeletonised with ligation of the right gastric artery. Portal lymphadenectomy is completed, and the gastoduodenal artery (GDA) is dissected free. The GDA is clamped to ensure adequate flow in the common hepatic artery and exclude retrograde flow to the hepatic vasculature via the SMA because of coeliac artery stenosis. If adequate flow is present in the hepatic artery, then the GDA is ligated and divided. The common hepatic artery lymph node can be resected en bloc at this point. In general, coeliac and SMA lymphadenectomy are only performed if there is clear evidence of lymphadenopathy. This clearly has an impact upon patient morbidity and survival after pancreatic surgery. Of note, extended lymphadenectomy offers no survival benefit to patients but increases surgical complications [21]. In our unit, CT-PET is performed on all patients considered for pancreatic surgery and may be able to inform on the utility of this dissection [22]. During the dissection of hepatoduodenal ligament, care must be taken to avoid injury to accessory vessels and preoperative imaging in the form triple-phase CT may assist the surgeon in this phase of the operation [23] (Figure 2). At this point, the retropancreatic tunnel can be completed and a nylon tape passed behind the pancreas and in front of the portal vein/SMV.

The stomach (classical Whipple) or duodenums (PPPD) are now divided with surgical stapling devices followed by the stapling and division of the jejunum. If the duodenum and pylorus have been compromised by a tumour and/or inflammation, then a classical Whipple is performed. The jejunal mesentery is divided using an appropriate energy device ensuring no injury occurs to the inferior mesenteric vessels. The jejunum can then be delivered under the superior mesenteric vessels and into the surgical field. Once this has been achieved, the pancreatic neck can be divided with either a knife or diathermy. This now allows the uncinate process of the pancreas to be dissected free from the SMV using ligatures and/or clips to control small venous tributaries. The inferior (IPDA) and superior (SPDA) pancreaticoduodenal arteries are also controlled and divided during this phase of resection. The remaining retroportal lymphadenectomy is completed with the skeletonisation of the lateral border of the SMA. This latter dissection has already been performed if the posterior artery-first approach has been utilised. The end result at the conclusion of the resection will appear similar to Figure 2.

**Figure 3:**
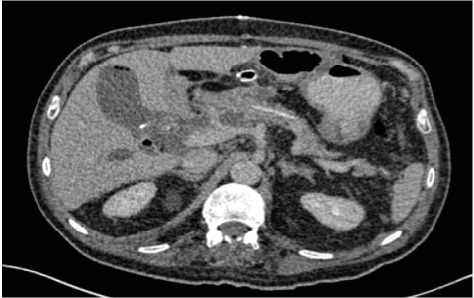
Postoperative CT demonstrating a single layer pancreaticojejunostomy using a dunking technique performed over a pancreatic duct stent.

Following pancreatic resection, the reconstructive phase of surgery commences and begins with pancreatic resection. There is little clinical evidence to support pancreatico-jejunostomy (PJ) over pancreatico-gastrotomy (PG) reconstruction, but a recent meta-analysis clearly demonstrated that most surgeons' preference is to perform a P-J anastomosis [24]. This area is controversial as the PJ anastomosis can be performed using a variety of methods. The PANasta Trial is attempting to clarify this area although it is only evaluating duct-to-mucosa PJ anastomosis techniques (Cattell and Blumgart techniques) and not other methods of pancreatic anastomosis such as the dunking method that is the preferred method for, at least, the authors on this manuscript [25]. There is emerging evidence that pancreatic duct diameter combined with gland texture can predict the incidence of postoperative pancreatic fistula (POPF) in addition to the PJ anastomosis technique used [26]. In addition, the authors use a pancreatic stenting during reconstruction routinely with a reported benefit in reduced pancreatic fistula incidence [27] (Figure 3).

Following this, end-to-side, single-layer, interrupted, or single-layer continuous hepaticojejunostomy with or without stenting is performed using the same jejunal loop. The decision as to the mode of reconstruction is usually dependent upon the bile duct caliber. It is the author's preference to perform a double-layer, continuous, hand-sewn antecolic gastrojejunostomy or duodenojejunostomy, with a nasogastric tube placed in the stomach and a feeding nasojejunal tube in the efferent jejunal limb of the anastomosis for postoperative nutrition. The authors advocate encircling the earlier mobilised falciform ligament around the pancreatic anastomosis to reduce the risk of GDA pseudoaneurysm. The postoperative management of pancreatic resections is outside the scope of this manuscript, but the reader is referred to recent excellent review article [28].

In a recent single center study, Picozzi et al. reported a 5-year survival of patients with resected PDAC of 32% and overall survival of 34% [29]. Whilst these results are encouraging, they still far short of the survival rates for other resected cancers. As noted in the above study, 50% of the patients developed systemic disease after resection for PDAC [29]. To address this, many groups are assessing the role of neoadjuvant chemotherapy in setting of resectable PDAC such as the NorPACT-1 Trial [30] with recent meta-analysis demonstrating that multiagent neoadjuvant chemotherapy is associated with improved survival in patients undergoing pancreatic resection [31]. Although as suggested by Heinrich et al., despite supporting evidence of the treatment effect neoadjuvant therapy, it has not proven superior to surgery in randomised trials for resectable PDAC with most available data based upon small phase II trials [32]. To add further weight to this latter point, Zhan et al. recent meta-analysis of both resectable, borderline resectable, and locally advanced PDAC suggested that whilst it may offer benefit to the patients in the latter two groups, it should be used with caution in patient with resectable PDAC as it was not shown to be beneficial [33]. Much of this discordant surgical data may in part be due to the type of chemotherapy used as Unno et al. demonstrate that a combination of gemcitabine and S1 in resectable PDAC significantly improved overall survival (36.7 months versus 26.6 months (upfront surgery) [34]. This area of PDAC management is likely to remain a fertile area of research especially as more sophisticated chemotherapy agents are developed and the results of the NorPACT-1 may demonstrate that neoadjuvant chemotherapy is beneficial in resectable PDAC in a randomised trial setting. The results of such trials are eagerly awaited.

## 4. Artery-First Approach to Pancreatic Resection

**Figure 4:**
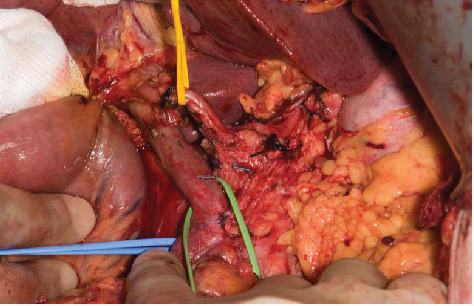
The posterior artery-first approach for the pancreatic cancer. The pancreatic resection has been completed. The blue sloop is encircling the SMA and the green sloop the portal vein. All the retropancreatic tissues have been divided prior to pancreatic neck division. Gerota's fascia is apparent, and the yellow sloop is displacing the common hepatic artery. This patient had also received neoadjuvant chemoradiation.

The artery-first technique for pancreatic resection has been excellently summarised by Sanjay et al. [35]. Most pancreatic surgeons are trained or prefer the approach originally described by Marzano et al. and is referred to as the “posterior approach” [36] (Figure 4).

Following Cattell-Braasch manoeuvre that has been detailed above, the root of the SMA is dissected and exposed where it passes in front of the left renal vein in front of the abdominal aorta. The perineural and perivascular tissue can be carefully divided dissecting away from the abdominal aorta, posterior to the pancreatic head aiming towards the duodenum. As this dissection continues, tissue between the SMA and uncinated process are progressively divided to expose the lateral border of the portal vein/SMV. This allows a relatively easy access to the IPDA and SPDA for ligation. Data demonstrate that this dissection enables assessment of vascular involvement and also allow safe dissection of accessory/replaced right hepatic artery (Figure 2) whilst also reducing blood loss during surgery [37–39]. Aside from this approach, the medial uncinate approach, inferior infracolic approach, the left posterior approach, inferior supracolic approach, and superior approach have also been described [35].

The decision to perform total pancreatectomy may have been made preoperatively because of the presence of multifocal disease or, in some instance, be made intraoperatively because of vascular involvement thereby performed to ensure oncological clearance (see below). Additionally, positive resection margins on frozen sections sent during surgery may necessitate total pancreatectomy. The operative approach for total pancreatectomy is as described above. Dependent upon surgeon preference, an artery-first approach can be utilised or a more conventional approach be adopted. We recommend posterior artery-first approach when performing a total pancreatectomy as this firstly allows skeletonisation of the SMA and without pancreatic neck division, as in a Whipple/PPPD, allows division for the retropancreatic tissue and mobilisation of the uncinate process ensuring that the correct plane posterior to the pancreatic body and tail is entered. Following the division of this tissue, the tortuous splenic artery is dissected free and controlled with a sloop until division, followed by isolation of the splenic vein. Short gastric vessels can be controlled with a suitable energy device and the stomach reflected cranially. By adopting the artery-first approach, the SMA now lies outside the dissection plane and adrenal gland along with Gerota's fascia is used as the posterior extent of resection of the left side of the abdomen. At this point, the splenic artery, splenic vein, and the inferior mesenteric vein can be ligated. The spleen is included in the resection for oncological reasons, and thus, the splenorenal ligament is divided and drawn medially to meet the plane developed by the dissection posterior to the pancreatic body and tail. The duodenum/stomach and jejunum are mobilised and transected as described above followed by en bloc resection of the pancreas, duodenum, spleen, and the peripancreatic lymph nodes. Total pancreatectomy eliminates the need for a pancreatic reconstruction anastomosis but causes iatrogenic diabetes mellitus, which maybe brittle, and exocrine insufficiency [40]. However, in selected cases, total pancreatectomy can ensure R0 resections with comparable patient outcome to Whipple/PPPD [41].

## 5. Pancreatic Resection in the Presence of Vascular Involvement

As eluded to above, pancreatic surgery is evolving with surgical approaches being modified in patients who have received neoadjuvant treatment [42–44]. These preoperative regimens are principally being used for patients with borderline resectable or locally advanced PDAC [45], although the use of these strategies is attracting growing attention in patients with resectable disease, too [42], as discussed above [30]. There are multiple definitions of borderline resectable and locally advanced PDAC from many learned global societies (Table 1).

**Figure 5:**
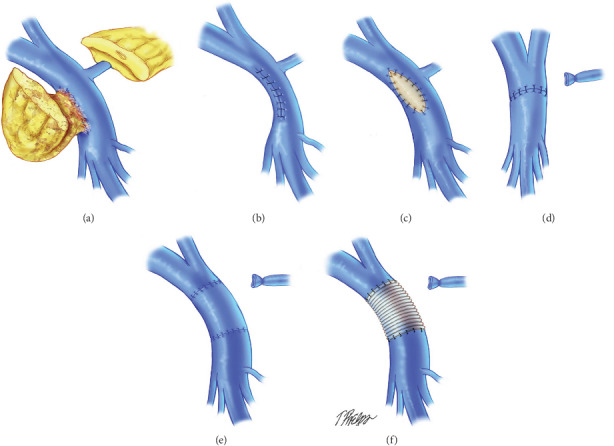
Surgical methods for vein reconstruction after vein resection for PDAC. (Taken from Glebova et al., J Vasc Surg 622: 424-443).

Surgery after neoadjuvant regimens for PDAC presents challenges to the pancreatic surgeon. Preoperative radiation especially causes localised inflammation that changes the surgical field by inducing adhesions particularly between the tumour and adjacent organs such the colon, stomach, and IVC. A particular concern for the surgeon is adherence between the tumour and the portal vein/SMV. Clearly, if this cannot be dissected freely, then vein resection needs to be considered. The radiation field will also encompass the porta hepatitis which results in fibrosis with again the loss of normal tissue planes as demonstrated in Figures 2 and 4. Whilst possible via a rooftop incision, a midline incision with Cattel-Braasch manoeuvre as described above is considered the optimal approach in patients where vein resection is likely to be needed. The posterior artery-first approach described above can be difficult to achieve due to inflammation and/or fibrosis present at the root of the mesentery. There is merit in pursuing this approach as if the SMA can be dissected freely and mobilised away from the tumour, then resectability of the tumour has been proved and a vein resection can be carried out then if needed. Whether this approach is adopted or not, the pancreas must be dissected freely from the splenic vein to the left of the portal vein/SMV junction to facilitate safe division of the pancreas to the left of the pancreatic neck. Prior to pancreatic division, adequate venous clearance is required to enable safe clamping of the veins prior to vein resection. The portal vein is dissected free up to its bifurcation into the left and right portal veins and the SMV dissected free to its first order branches, although if a safe plane can be cleared above the SMV branches, this maybe satisfactory for vein clamping. Once this has been achieved, the patient is heparinized with an unfractionated heparin bolus. The already slooped portal vein, splenic vein, and SMV are all clamped with appropriate vascular clamps. The pancreas is divided, followed by division of the SMV and portal and splenic vein (if required), and then the surgical specimen is removed. The advantage of having previously mobilised the mesentery using the Cattell-Braasch manoeuvre in this scenario is that if a complete vein resection is required, a defect of up to 4 cm can be reconstructed using an end-to-end venous anastomosis thus precluding the need for interposition venous graft (Figure 5(d)), meaning that pancreatic resection with full-vein reconstruction can be safely performed outside transplantation units [46].

In most patients with venous involvement, a wedge of the portal vein or SMV can be performed if a small portion of the pancreatic head mass is inseparable from a vein by applying a Satinsky vascular clamp to the appropriate area of the vein and closuring the defect with fine Prolene sutures (Figure 5(b)). For segmental resections of the portal vein or the SMV longer than 4 cm, end-to-end anastomosis can be performed with the use of a vascular conduit for reconstruction (Figure 5). Appropriate conduits include autologous vein (left renal vein, great saphenous vein, or internal jugular vein (Figure 5(d)), cryopreserved cadaveric vein or artificial gortex grafts (Figure 5(f)), although vein grafts have an increased risk of late venous thrombosis [47–49]. The anastomosis is usually preformed using a continuous single layer 6/0 Prolene suture with an appropriate growth factor. The remainder of the operation including reconstruction is completed as detailed above.

The first randomised controlled trial using neoadjuvant regimens for borderline resectable PDAC has demonstrated that patients in the neoadjuvant arm derive such a significant survival benefit that the trial was closed early [50]. In this trial, a resection rate of 63% was reported following neoadjuvant chemoradiation, although vascular reconstruction rates were not reported. Although in a recent study from John Hopkins Institute, the need for vein resection was reported as 49% in patients undergoing pancreatic resection following neoadjuvant treatment [51]. In addition, Dhir et al.'s recent meta-analysis of 5520 patients with resected PDAC demonstrated neoadjuvant treatment increases in R0 resection rates and has equivalent survival in the resectable versus borderline resectable PDAC cohorts after surgery [52]. Although arterial resection and reconstruction has been reported in patients with PDAC, this is currently not favoured because of the high risk of patient mortality [53]. In summary, the literature suggests that pancreatic resection with or without vein resection in locally advanced and borderline resectable PDAC following neoadjuvant treatment appears safe and derives survival benefit for the patient.

## 6. Minimally Invasive Pancreatic Resections for PDAC

**Figure 6:**
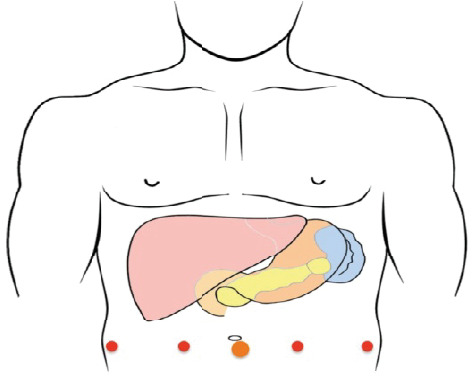
A 12 mm port is placed two fingerbreadths below and to the right of the umbilicus, two robotic ports are placed in the right upper quadrant, a port is placed in the right lower quadrant, a port in the left lower quadrant, and a port in the anterior axillary line on the left side of the abdomen.

Minimally invasive pancreatic surgery for PDAC (either laparoscopic or robotic) aims to give the same oncological outcomes as open surgery but negate the need for a large abdominal incision. Whilst surgeons have a longer experience with laparoscopic pancreatic resection, robotic platforms are gaining momentum as a viable option for patients requiring pancreatic resections. Robotic platforms, in addition, offer technical advances that aid in the performance complex surgical procedures such as Whipple/PPPD. Indeed, with the use of such facets as 3-D viewing and EndoWrist technology, encouraging early results have been reported with robotic Whipple/PPPD [11]. The procedure of robotic pancreaticoduodenectomy (RPD) remains in its infancy, and a standard stepwise approach to this surgery is not well described in the literature. Similarly, laparoscopic pancreaticoduodenectomy (LPD) is performed globally but a standard approach remains to be agreed internationally. In general, for either RPD or LPD, the patient is placed in a supine position with reverse Trendelenburg positioning. For LPD, between 5 and 6 trocars can be used, but for RPD 4, robotic arms can be used on the platform with two assistant ports [54]. The placement for robotic PPPD is demonstrated in Figure 6.

For both RPD and LPD, the peritoneal cavity is generally accessed using an open (Hassan) technique as indicated in Figure 6. The abdominal cavity is insufflated with CO2 gas, and as in open surgery, the presence of metastatic disease is excluded. The remaining working ports are then inserted. Variations on the port placement arrangement demonstrated in Figure 6 are common. As for many upper abdominal surgical procedures, a liver retractor is placed and the liver retracted in a cranial direction. Although intuitively the approach to minimal access is markedly different to open surgery, once the liver is retracted, the ligament of Treitz is identified and a marker suture is placed 70-80 cm distal to the duodenal-jejunal flexure. This is a critical step as once pancreatic resection is completed, it may become extremely difficult to assess the optimal position for the gastrojejunostomy, and this suture aids in this decision-making process. The patient is now manoeuvred into a steep head-up position with the right side up in order to allow the small bowel to migrate to the lower abdominal cavity. This is akin to the position of patients undergoing bariatric surgery. If a RPD is being performed, then the robotic platform is docked and remaining instruments are docked to the robot arms.

In contrast to the open approach described above in LPD/RPD, the greater omentum is opened below the gastroepiploic pedicle to gain access to the lesser sac and hence the pancreas. The greater curvature omentum is mobilised by employing a minimal access energy device. The transverse colon and hepatic flexure can now be mobilised, and the colonic mesentery is dissected off Gerota's fascia allowing the surgeon access to the duodenum and pancreatic head. Once this point in the dissection has been reached, Kocherization can be performed with skeletonisation of the IVC and left renal vein. Akin to the open approach, the ligament of Treitz can be fully mobilised. The duodenum and jejunum can be divided with a suitable stapling device, and the jejunum can be transposed to the right side of the abdomen. Clearly, this is a committal step, and hence, most experience with the minimal access approach involves PDAC that does not compromise the major vasculature or pancreatic neck. A cholecystectomy can be performed and the common bile duct divided. The hilar lymphadenectomy can be completed with attendant care to the hepatic artery. It remains to be demonstrated that these approaches provide similar lymphadenectomy to open approach. The next phase of the dissection is similar to the open approach with GDA division and completion of the retropancreatic tunnel that is fashioned from the inferior pancreatic border to the superior aspect. A nylon tape can be passed and an assistant port used to retract the pancreas towards the abdominal wall with the pancreas divided with diathermy. In an analogous manner to open surgery, the uncinate process, venous tributaries, and IPDA/SPDA are controlled with a combination of energy devices and metallic clips. The surgical specimen can now be placed in a sterile bag.

The proximal jejunum is transferred to the supracolic compartment lateral to the middle colic vessels through a mesocolic defect. As with the open approach, there are a number of methods for minimal access reconstruction of the pancreatic remnant with or without a stent. Most minimal access pancreatic surgeons perform the hepaticojejunostomy using self-locking V-Loc sutures. The gastrojejunostomy can be fashioned using sutured or stapled methods. Abdominal drainage is as per open surgery.

Although not based upon randomised data, retrospective series suggests that there is an advantage to performing minimal access pancreatic surgery in the form of RPD [55]. It is reported that once a surgeon has completed the learning phase of RPD, total operative time, in-patient stay, and estimated blood loss can be lower than those in open surgery and at least equivalent to LPD [56]. In addition, the rates of POPF and other serious complications following RPD were reported at lower frequencies [56]. In studies that have compared RPD to open Whipple/PPPD in a prospective manner, RPD was reported to have a longer operative time but faster return-to-functional recovery [57]. Importantly, the two groups had similar reported rates of surgical morbidities and mortality, R0 resection rates, and overall and disease-free survival. Furthermore, recent analysis of RPD versus LPD demonstrated that R0 resection rates of 100% with acceptable postoperative complications [58].

Laparoscopic and robotic total pancreatectomy remains in their infancy, although there are an increasing numbers of single surgeon reports. This is likely because many of the as-yet unresolved challenges with open pancreatic surgery, such as the optimal mode of pancreatic reconstruction, are not addressed by the minimal access approach. Indeed, minimally invasive pancreatic resection for borderline resectable disease is not yet recommended and open surgery remains the standard approach here.

## 7. Minimally Invasive PPPD with Vascular Involvement

There are very few reports of successful minimal access resection of PDAC in the presence of vascular involvement. As with open surgery in patients with vascular involvement, the preferred approach would be an artery-first approach to prove resectability. However, without tactile feedback, in the case of robotic surgery and potentially limited views with laparoscopic surgery, such cases are technically demanding and hence not commonly performed. In such patients, most surgeons will opt to perform open surgery. The exception to this is if venous involvement is limited and resection can be achieved with primary vein repair, but again, if this feature was evident on preoperative imaging or noted intraoperatively, most surgeons would adopt an open approach.

## 8. Distal Pancreatectomy and Splenectomy (DPS)

For PDAC that is located in the pancreatic body and tail, minimally invasive DPS is now accepted as the standard of care [59]. A number of factors have contributed to the surgical community adopting this practice. Principally, the lack of a pancreatic anastomosis means that the procedure is associated with reduced levels of morbidity and mortality and additionally, the learning curve for the procedure is of lesser magnitude than the operations discussed above [60]. Whilst the laparoscopic DPS is practiced by most surgeons when indicated, robotic DPS is being increasingly performed but further oncological data is required before it rivals laparoscopic DPS as the standard of care [61].

Minimal access DPS can be performed through a variety of closely aligned methods. Generally, whether performed by the laparoscopic or robotic approach, 4 to 5 port trocars are placed in a semicircular fashion centered on a periumbilical optical port. Similar to minimal access Whipple surgery, a liver retractor is used to reflect the left lobe cranially. The gastrocolic ligament is opened with an energy device, while preserving the gastroepiploic vessels. Once this is achieved, the liver retractor is repositioned to retract the stomach and left lobe of the liver cranially allowing access to the pancreatic tail and spleen. Although intraoperative imaging can be used to locate the tumour, lesions in the pancreatic body or tail are usually macroscopically visible. In the conventional approach, the inferior border of the pancreas is defined with adequate mobilisation such that the splenic vein can be visualised allowing the dissection to continue posterior to it. With an adequate plane developed, the superior border of the pancreas is defined with isolation and control of the splenic artery. At this point, it is safe to pass a nylon tape around the pancreatic body/tail. Fine vessels entering the posterior pancreas can usually be controlled with an appropriate energy device. The splenic artery and vein can be divided after application of surgical clips or Hem-o-loc® clips. The pancreas can then be divided with a stapling device, the spleen is then mobilised in a similar manner to that described above for total pancreatectomy with splenectomy, and the DPS is resected en bloc.

Despite this well-described surgical approach, the patient survival after DPS for PDAC is poor [62]. To improve on these disappointing patient outcomes, the radical antegrade modular pancreaticosplenectomy (RAMPS) was developed by Strasberg et al. The premise of this procedure being that the DPS described above does not provide an adequate or radical lymphadenectomy. Thus, although the initial approach is similar between DPS and RAMPS, once the surgeon enters the plane posterior to the pancreas in the RAMPS, the plane is carried deeper to include Gerota's fascia and the adjacent lymph nodes ensuring a more radical lymphadenectomy. The RAMPS can then entail resection of the left adrenal gland (posterior RAMPS) or not (anterior RAMPS) [63]. A key feature of the RAMPS procedure is the additional lymphadenectomy along the coeliac trunk. There is limited data as to the oncological outcomes for patients following RAMPS for PDAC, the anatomical logical of the approach are undoubtedly sound. Indeed, a recent meta-analysis demonstrated that minimally invasive DPS had comparable 5-year survival to open surgery with significantly lower positive margin rates, shorter in-patient hospital stay, less blood loss, and lower patient morbidity and mortality [64]. As discussed above, the data on RAMPS is limited by analysis of retrospective data which demonstrate that there is no increase in POPF, complications, and mortality but a significantly improved R0 rate and number of lymph nodes resected [65]. Although as Cao et al. state further, trial data are needed before confirmation of the survival benefits of RAMPS which can be assessed although the authors state that based upon their meta-analysis, RAMPS is an oncologically superior procedure [66]. Finally, the use of robotic DPS/RAMPS for PDAC may allow further advances in the radicality of dissection and regional lymphadenectomy although robust data on this is required [67].

## 9. Summary

Many surgical approaches are available when planning resection of PDAC. In general, when vascular resection and reconstruction are required, the open approach appears most feasible whilst in those patients with early disease, the minimal access/robotic approach appear to offer improved patient metrics without apparent comprise on oncological outcomes.

## Figures and Tables

**Table 1:** Different international classifications of locally advanced and borderline resectable PDAC (taken by JA Pietryga and DE Morgan (2015), Journal of Gastrointestinal Oncology, vol. 6, no. 4).

Anatomy	NCCN 2014	AHPBA/SSAT/SSO	MD Anderson Cancer Center	ISGPS	ACTO
Superior mesenteric vein/portal vein	Involvement with distortion/narrowing and/or occlusion amenable to reconstruction	Abutment encasement or short-segment occlusion amenable to reconstruction	Short-segment occlusion amenable to reconstruction	Involvement with distortion/narrowing and/or occlusion amenable to reconstruction	Tumor-vessel interface ≥180° and/or occlusion amenable to reconstruction
Superior mesenteric artery	Abutment (≤180°)	Abutment (≤180°)	Abutment (≤180°)	Abutment (≤180°)	Tumor-vessel interface <180°
Common hepatic artery	Abutment or short-segment encasement	Abutment or short-segment encasement	Short-segment encasement/abutment	Abutment or short-segment encasement	Short-segment tumor-vessel interface (any degree) amenable to reconstruction
Celiac artery	No abutment or encasement	No abutment/encasement	No abutment or encasement	No abutment or encasement	Tumor-vessel interface <180°

NCCN: National Comprehensive Cancer Network; AHPBA/SSAT/SSO: American Hepato-Pancreato-Biliary Association/Society for Surgery of the Alimentary Tract/Society of Surgical Oncology; ISGPS: International Study Group of Pancreatic Surgery; ACTO: Alliance for Clinical Trials in Oncology.
